# Aberrant expression of cyclin A in head and neck cancer

**DOI:** 10.1054/bjoc.2000.1193

**Published:** 2000-06-02

**Authors:** M Lotayef, G D Wilson, F M Daley, H K Awwad, T Shouman

**Affiliations:** Gray Laboratory Cancer Research Trust, Mount Vernon Hospital, Northwood, Middlesex, HA6 2JR, UK; National Cancer Institute, Cairo University, Fom el Khalig, Cairo, Egypt

**Keywords:** cyclin A, proliferation, head and neck cancer

## Abstract

Cyclin A expression was studied in a series of 65 squamous cell carcinomas of the head and neck (HNSCC) and compared with known markers of proliferation, iododeoxyuridine (IdUrd) and Ki-67, to assess whether aberrant expression was prevalent. Patients had previously been administered IdUrd to study cell kinetics in relation to outcome of radiotherapy. The data showed that all three parameters were highly correlated although the absolute values were different. The median labelling indices (LI) for IdUrd, cyclin A and Ki-67 were 10.7, 17.1 and 30.8% respectively, reflecting the known pattern of differential cell cycle expression. However, there were a significant number of cases in which an unexpected relationship between cyclin A and either IdUrd or Ki-67 was present. Some of these were attributable to overexpression but others indicated underexpression. Although the greater variability and range of cyclin A expression, coupled with its more closely associated role in cell cycle regulation, might suggest that it may be a more informative marker for cell proliferation than Ki-67, the aberrant expression seen in over one third of cases would indicate that caution should be exercised in interpreting cyclin A as a surrogate marker of proliferation in HNSCC. © 2000 Cancer Research Campaign


*British Journal of Cancer* (2000) **83**(1), 30–34
© 2000 Cancer Research Campaign
doi: 10.1054/ bjoc.2000.1193, available online at http://www.idealibrary.com on IDEAL®

### Aberrant expression of cyclin A in head and neck cancer

**M Lotayef[1], GD Wilson[1], FM Daley[1], HK Awwad[2] and T Shouman[2]**

[1]Gray Laboratory Cancer Research Trust, Mount Vernon Hospital, Northwood, Middlesex HA6 2JR, UK; [2]National Cancer Institute, Cairo University, Fom el Khalig, Cairo, Egypt

**Summary** Cyclin A expression was studied in a series of 65 squamous cell carcinomas of the head and neck (HNSCC) and compared with known markers of proliferation, iododeoxyuridine (IdUrd) and Ki-67, to assess whether aberrant expression was prevalent. Patients had previously been administered IdUrd to study cell kinetics in relation to outcome of radiotherapy. The data showed that all three parameters were highly correlated although the absolute values were different. The median labelling indices (LI) for IdUrd, cyclin A and Ki-67 were 10.7, 17.1 and 30.8% respectively, reflecting the known pattern of differential cell cycle expression. However, there were a significant number of cases in which an unexpected relationship between cyclin A and either IdUrd or Ki-67 was present. Some of these were attributable to overexpression but others indicated underexpression. Although the greater variability and range of cyclin A expression, coupled with its more closely associated role in cell cycle regulation, might suggest that it may be a more informative marker for cell proliferation than Ki-67, the aberrant expression seen in over one third of cases would indicate that caution should be exercised in interpreting cyclin A as a surrogate marker of proliferation in HNSCC. © 2000 Cancer Research Campaign

**Keywords:** cyclin A; proliferation; head and neck cancer

Deregulation of the cell cycle control machinery is a frequent event in cancer (Sherr, 1996). The majority of oncogenes and tumour suppressor genes function in signal transduction to mimic the effects of persistent mitogenic stimulation. Overcoming the regulatory influences of extracellular signals facilitates continuous cell cycling (Pardee, 1989). Yet, solid tumours exhibit considerable heterogeneity in cellular proliferation characteristics that represent the final manifestation of these changes (Dolbeare, 1995).

Although certain generalizations can be made concerning the overexpression or loss of function of a particular gene in terms of its likely influence on cellular proliferation, the relationship is not always obvious. Finding reliable and informative markers of cellular proliferation is still of great importance for clinical studies as both prognostic and predictive markers of outcome in various tumour types.

In recent years, the identification and characterization of a number of cell cycle-associated proteins, particularly cyclins, cyclin-dependent kinases and their positive and negative regulators (Pines, 1995), has opened up new possibilities for proliferation markers. Of these, the cyclins would appear to hold most promise to become a surrogate marker for proliferation due to their association with specific cell cycle checkpoints and phases (Bodey et al, 1994; Dutta et al, 1995). The caveat to this putative role is whether cyclins are acting as proto-oncogenes resulting in inappropriate expression in tumours. This is clearly the case for cyclin D1 (Hall and Peters, 1996) and a similar role has been suggested in some studies for cyclin A (Furihata et al, 1996; Wang et al, 1990).

However, to establish true overexpression there needs to be comparison made with known markers of proliferation and preferably with multiple markers associated with different cell cycle phases (Dutta et al, 1995; Handa et al, 1999). In this study we took advantage of a cohort of patients with HNSCC who had been administered IdUrd to measure potential doubling time ($T_{pot}$) in a study of radical and postoperative radiotherapy. Using immunohistochemistry, we were able to stain IdUrd positivity to delineate the S-phase and utilized Ki-67 expression to measure the approximate growth fraction. This enabled the evaluation of cyclin A as marker associated with S+ G2 phase and the capability of resolving whether inappropriate expression was present.

### MATERIALS AND METHODS

### Patients

The patients were drawn from a prospective study of locally advanced squamous cell carcinoma of the head and neck treated with surgery and postoperative radiotherapy or with radical radiotherapy only. These patients (47 males, 18 females, ages 22–68) were treated in the National Cancer Institute, Cairo University during the period between 1990 and 1997 for the postoperative group and between 1994 and 1997 for the radical group. All patients were previously untreated, age less than 65 years, performance status 0, 1 or 2 according to the WHO scale and had no evidence of distant metastasis or second primary malignancy. All specimens were taken from the primary tumour (31 oropharynx, 13 hypopharynx and 21 larynx) either at biopsy (33 patients treated by radical radiotherapy) or resection (32 patients treated by

*Received 28 October 1999*
*Revised 16 February 2000*
*Accepted 24 February 2000*

*Correspondence to*: GD Wilson postoperative radiotherapy) and assessed by a pathologist. Twelve tumours were classified as well differentiated, 42 were moderately differentiated and 11 were poorly differentiated. There were 15 T2 tumours, 29 T3 and 21 T4. Thirty-seven tumours were without nodal involvement, while 18, 8 and 2 were N1, N2 and N3 respectively.

### Iododeoxyuridine

5-iodo-2′-deoxyuridine was obtained as a freeze-dried powder in 250 mg vials (NCI Investigational Drugs Brach, Bethesda, USA). Immediately prior to injection, the drug was dissolved in 10 ml of 0.9% NaCl and then passed through a bacterial filter into a sterilized vial. The drug was injected 4–6 h before biopsy as an intravenous bolus in patients who were randomized for radical radiotherapy, or 4–6 h before surgery in patients who were randomized for surgery and postoperative radiotherapy. Specimens were examined by a pathologist and pieces were either fixed in 10% buffered formalin for immunohistochemistry or 70% ethanol for flow cytometry.

### Immunohistochemistry

Each of the three proliferation markers was stained using the same method, with the exception that the antibody against Ki-67 was not monoclonal and derived from rabbit. Briefly, 4 μm sections were taken to 70% alcohol and endogenous peroxidase blocked for 30 min in methanol containing 1% $H_2O_2$. The sections were pretreated by 4 × 4 min microwave irradiation in 10 mM citrate acid. After blocking with 1:5 normal swine serum (for Ki-67 only), the sections were incubated with either 1:200 dilution of rabbit anti-Ki-67 antibody (Dako Ltd, High Wycombe, UK) or 1:75 dilution of mouse anti-BrdUrd/IdUrd antibody (Dako Ltd) or a 1:50 dilution of mouse anti-cyclin A antibody (Novocastro, Newcastle, UK) in TBS. Incubations were either 2 h at room temperature or overnight at 4°C. Staining was visualized using swine anti-mouse or rabbit secondary antibody (Dako Ltd) diluted 1:350 in TBS and incubated at room temperature for 1 h. ABComplex (Dako Ltd) was then added for 1 h at room temperature and then DAB solution for 10 min. The sections were counterstained with haematoxylin, dehydrated mounted in DPX.

### Assessment of labelling

The labelling of each marker was assessed in serial sections from each tumour. Exact fields were not matched between each lable due to the difficulty of identifying landmark features. However, the same procedure of selecting alternating transverse and longitudinal fields was used. There was no attempt to select fields of maximum labelling. The method of quantitation involved capturing images of between 9 and 20 high-power fields (× 40 objective × 10 eyepiece) fields using a Zeiss Axioskop trans-illumination microscope, coupled to a JVC KY55F 3-CCD colour camera.

Images were grabbed by digitizing them using a Matrox Meteor frame grabber, installed in a PCI bus machine. The images were analysed using a manual routine within the Visilog (Noesis SA) software package program. Labelling was assessed by marking each positive and each negative tumour nuclei in each high-power field, to obtain a minimum of 1000 cells per specimen. The LI was calculated as the total number of IdUrd, Ki-67 or cyclin A-labelled cells vs the total tumour cells counted. A correction was made for the IdUrd LI as the total count does not take into account cell division between injection and biopsy. A correction was made by consideration of the flow cytometry data, in which the fraction of the IdUrd labelled cells that had divided or remained undivided was known. A simple proportion from uncorrected and corrected FCM LI was used to correct the immunohistochemistry LI.

### Flow cytometry

The duration of S-phase (Ts) was calculated for each specimen using the method of Begg et al (1985) which permits the estimation of Ts from a single biopsy taken several hours after the injection of IdUrd; the details of this method have been described elsewhere (Wilson, 1998).

### Statistical analysis

A variety of statistical analyses were carried out using the JMP package (SAS Institute, Cary, NC, USA), these included Spearman's rank correlation, Mahalanobis outliner analysis and Wilcoxon and Kruskal Wallis analysis of significance.

### RESULTS

### Immunohistochemical data

From the 65 specimens a total of 888, 931 and 710 fields were captured and counted for IdUrd, Ki-67 and cyclin A respectively. Within these fields, the overall median values, ranges and CVs for the three markers are shown in Table 1, as are the same data calculated on an interpatient median value basis. It can be seen that the median values follow the presupposed cell cycle pattern with IdUrd giving the lowest positivity followed by cyclin A and finally Ki-67. Each of the markers was significantly different to each other ($P > 0.0001$).

### Correlation analysis

Spearman's rank analysis was carried out to correlate the three markers. Table 2 shows that there were highly significant correlations

**Table 1** Comparison of the proliferation markers. The data are presented as the median, range and CV for each marker as a percentage. All fields refers to complete data set whilst intertumour values were calculated from the median values for each of the 65 specimens

| | Idurd | | | Ki-67 | | | Cyclin A | | |
|---|---|---|---|---|---|---|---|---|---|
| All fields | 11.5 | 0–68.6 | 61.7 | 31.3 | 2.8–82.6 | 44.6 | 19.0 | 1.0–79.3 | 52.7 |
| Intertumour median values | 10.2 | 3.2–26.7 | 41.9 | 30.8 | 8.8–56.8 | 37.3 | 17.1 | 4.7–38.7 | 45.6 |

© 2000 Cancer Research Campaign

**Table 2** Spearman's rank correlation analysis for the median values for each of the 65 patients

| Variable | By variable | Spearman Rho | Probability |
|---|---|---|---|
| Ki-67 | IdUrd | 0.433 | 0.0003 |
| Cyclin A | Idurd | 0.420 | 0.0005 |
| Ki-67 | Cyclin A | 0.356 | 0.0036 |

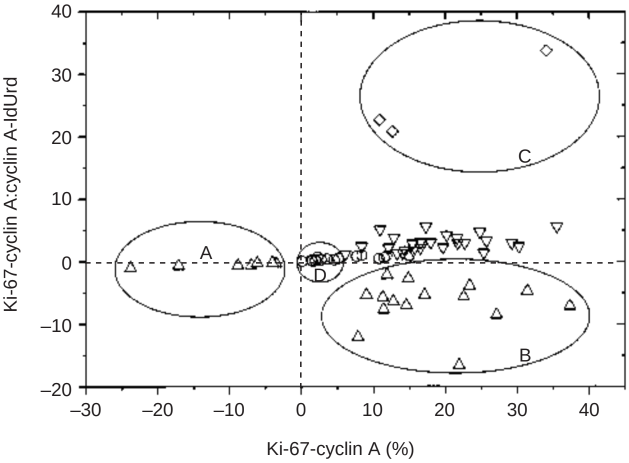

**Figure 1** The relationship between Ki-67, IdUrd and cyclin A expression in 65 HNSCC. The data are presented as a ratio of the difference between Ki-67 and cyclin A and cyclin A and IdUrd vs the difference between Ki-67 and cyclin A. This plot highlights subgroups who either overexpress or underexpress cyclin A (**A** and **B** respectively) or tumours in which cyclin A overexpression may be present (**D**) or three tumours in which cyclin A and IdUrd values were almost identical (**C**). The symbols are varied for clarity.

between the three markers. Comparisons involving IdUrd had the best correlation probabilities, while that between cyclin A and Ki-67 was the weakest. Correlation analysis was also carried out on the CV of the median values derived for each parameter from each patient. The reverse relationship became apparent. The inherent variability within specimens showed the best correlation between cyclin A and Ki-67 (Rho 0.409, $P = 0.0007$), followed by cyclin A and IdUrd (Rho 0.300, $P = 0.0152$) and finally the correlation between Ki-67 and IdUrd variability failed to reach significance (Rho 0.185, $P = 0.141$). There was no correlation between any of the parameters and the Ts values.

### Relationships between the markers and outlier analysis

To assess whether there was overexpression of cyclin A in some tumours, outlier analysis was used. The Mahalanobis outlier distance plot is useful when data are correlated, as it is possible for a point to be unremarkable when seen along one or two axes, but still be an outlier by violating the correlation. The analysis plots the Mahalanobis distance of each point from the multivariate mean (centroid). Interestingly, this analysis only revealed two outliers, one in which the IdUrd, cyclin A and Ki-67 values were 10.1, 32.6 and 8.8% respectively, and the other which had values of 26.7, 20.0, and 51.4%. However, visual examination of the data revealed several specimens that did not follow the expected pattern (Figure 1). The plot shows the various relationships and outliers that appear within the data set by plotting the difference between Ki-67 and cyclin A as a ratio of the differences between the three parameters (Ki-67-cyclin A/cyclin A-IdUrd). Seven tumours showed cyclin A values exceeding the Ki-67 value (cohort A in Figure 1).

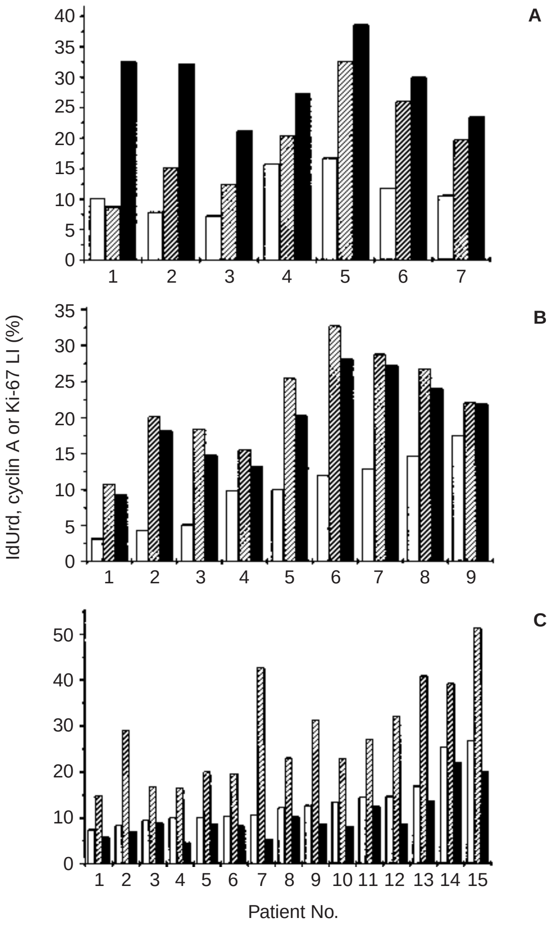

**Figure 2** Individual values for IdUrd (□), Ki-67 (▨) and cyclin A (■) in cohorts **A**, **B** and **D** defined in Figure 1.

Figure 2 shows the individual values for each parameter in this group of tumours. In these patients the Ki-67-cyclin A/cyclin A-IdUrd ratio is negative but approaches 0 because either the Ki-67 and IdUrd values were very close in two patients (1 and 4) or that the Ki-67 and cyclin A values were similar in five of the patients. In the latter group, the relationship between IdUrd and Ki-67 followed the expected pattern with at least a two-fold difference. In these patients, the increased cyclin A value may be attributable to overexpression and inappropriate positivity. Figure 1 also reveals other subgroups of tumours. Cohort B represents 15 tumours in which the cyclin A values were less than the IdUrd LI, thus producing a negative Ki-67-cyclin A/cyclin A-IdUrd ratio. The differences were not great (up to 6.7%, Figure 2) and these differences could be explained by counting errors or by a more restricted cell cycle expression of cyclin A. Cohort C comprises three tumours in which the cyclin A values only just exceeded the IdUrd value, thus the high positive ratio. Cohort D represents a group of nine tumours in which the cyclin A values were only 1–10% less than the Ki-67 values, while the IdUrd and Ki-67 relationship was as expected (Figure 2). These may also represent tumours in which aberrant expression of cyclin A was present, if the expected relationship between S+G2 and growth fraction is

 © 2000 Cancer Research Campaign taken into account. The other cases followed the expected pattern of cyclin A greater than the IdUrd score and thus the ratio tended towards positive values.

### Relationship with clinicopathological parameters

There was no correlation between any of the proliferation markers and tumour site, T-stage, N-stage or differentiation.

### DISCUSSION

Proliferation is a characteristic of all tumours; it is very rare to find a specimen in which proliferating cells cannot be detected. Yet studies in the literature report cyclin A-negative tumours (Furihata et al, 1996) and, conversely, of cyclin A overexpressing tumours. The former would seem unlikely considering the role of cyclin A in controlling the onset of DNA synthesis and its requirement for the G2-M transition. The latter is contentious unless comparisons with known proliferation markers are conducted. It is important to distinguish between increased expression associated with rapid proliferation, and increased expression resulting from gene amplification or post-translational modification, if cyclin A is to be used a proliferation marker or as another prognostic protein.

The clearest evidence of overexpression of cyclin A (and E) resulting from gene amplification or post-translational modification was reported in breast cancer patients by comparison with Ki-67 staining and S-phase fraction measured by flow cytometry (Dutta et al, 1995). In this study, there appeared to be a small cohort of five of 48 breast tumours which showed elevated cyclin A, which this increased to eight when cyclin E was also considered. Recently, Handa and colleagues (Handa et al, 1999) used both Ki-67 and in situ hybridization detection of histone H3 mRNA to compare with cyclin A expression in colorectal cancer. Neither of these two studies was ideal. In the former, S-phase fraction suffers from problems of dilution with diploid cells and in the latter, the data were scored semi-quantitatively.

The findings in this present study of head and neck cancer are in accord with the data in breast cancer (Dutta et al, 1995). We were able to demonstrate seven patients (11%) in which cyclin A exceeded the Ki-67 score. It should also be noted that there were a further nine patients in which cyclin A values were within 10% of the Ki-67 index. Considering the likely relationship between ‘growth fraction’ and S+G2, it cannot be discounted that these are also examples of aberrant expression. Therefore, up to 25% of HNSCC tumours may have evidence of overexpressed cyclin A with regard to their proliferative state. There was no indication of increased overall proliferation in these patients with respect to IdUrd and Ki-67; the median values were similar to the overall distribution. However, we also demonstrated tumours in which the cyclin A expression may have been less than expected with reference to IdUrd staining. These may be examples of a more restricted cell cycle distribution of cyclin A or of irregularity in cyclin synthesis. In addition, there was a trend for the Ts values of these tumours to be shorter (median 9.9 h vs 11.2 h overall).

The question that cannot be answered from this and other studies is whether cyclin A expression drives the proliferation characteristics of the tumour or is merely a consequence of proliferation. This is an important issue if reports continue to imply there is overexpression of cyclin A, whereas all that is being demonstrated is the expected increase associated with increased cell proliferation. In this present study, clear overexpression was not associated with increased proliferation. Alternatively, the influence of overexpression may be manifested in prognosis, as has been found in a number of other studies (Aaltomaa et al, 1999; Furihata et al, 1996; 1997 Handa et al, 1999; Kallakury et al, 1999). We have deliberately not reported the clinical outcome in this series, as patients were treated by both postoperative and radical therapy and by conventional, hyperfractionated and accelerated treatments. There is likely to be an influence of proliferation between these schedules but the numbers are too small in each group to be definitive. However, the overall local control and survival rates 60% and 65% respectively; all seven patients with aberrant overexpression of cyclin A achieved local control and are still alive.

In summary, these data provide evidence for a small cohort of patients with aberrant expression of cyclin A in HNSCC, the consequence of which was not reflected in increased proliferation, poorer prognosis or impaired survival. The role of cyclin A as an alternative marker of proliferation would be compromised in HNSCC without simultaneous knowledge of another proliferation marker such as Ki-67. However, the data emphasize the heterogeneity of cell cycle parameters and proliferation within HNSCC. There was not a clear-cut relationship between cyclin A expression and Ki-67 or IdUrd and this is likely to be reflected in both the biological behaviour of the tumours and their response to therapy. Whether the variability and unscheduled overexpression of cyclin A confers greater prognostic potential remains to be seen; we are currently assessing this marker in a study of 500 patients treated by conventional or accelerated fractionation.

### ACKNOWLEDGEMENTS

This work was supported by a grant from the Egyptian Cultural Bureau and the Cancer Research Campaign.

### REFERENCES

Aaltomaa S, Eskelinen M and Lipponen P (1999) Expression of cyclin A and D proteins in prostate cancer and their relation to clinopathological variables and patient survival. *Prostate* **38**: 175–182

Begg AC, McNally NJ, Shrieve DC and Karcher H (1985) A method to measure the duration of DNA synthesis and the potential doubling time from a single sample. *Cytometry* **6**: 620–626

Bodey B, Williams RT, Carbonaro Hall DA, Horvath A, Tolo VT, Luck JV Jr, Taylor CR and Hall FL (1994) Immunocytochemical detection of cyclin A and cyclin D in formalin-fixed, paraffin-embedded tissues: novel, pertinent markers of cell proliferation. *Mod Pathol* **7**: 846–852

Dolbeare F (1995) Bromodeoxyuridine: a diagnostic tool in biology and medicine, Part II: Oncology, chemotherapy and carcinogenesis. *Histochem J* **27**: 923–964

Dutta A, Chandra R, Leiter LM and Lester S (1995) Cyclins as markers of tumor proliferation: immunocytochemical studies in breast cancer. *Proc Natl Acad Sci USA* **92**: 5386–5390

Furihata M, Ishikawa T, Inoue A, Yoshikawa C, Sonobe H, Ohtsuki Y, Araki K and Ogoshi S (1996) Determination of the prognostic significance of unscheduled cyclin A overexpression in patients with esophageal squamous cell carcinoma. *Clin Cancer Res* **2**: 1781–1785

Furihata M, Ohtsuki Y, Sonobe H, Shuin T, Yamamoto A, Terao N and Kuwahara M (1997) Cyclin A overexpression in carcinoma of the renal pelvis and ureter including dysplasia: immunohistochemical findings in relation to prognosis. *Clinical Cancer Research* **3**: 1399–1404

Hall M and Peters G (1996) Genetic alterations of cyclins, cyclin-dependent kinases, and Cdk inhibitors in human cancer. *Adv Cancer Res* **68**: 67–108

Handa K, Yamakawa M, Takeda H, Kimura S and Takahashi T (1999) Expression of cell cycle markers in colorectal carcinoma: superiority of cyclin A as an indicator of poor prognosis. *Int J Cancer* **84**: 225–233

Kallakury BV, Sheehan CE, Rhee SJ, Fisher HA, Kaufman RP Jr, Rifkin MD and Ross JS (1999) The prognostic significance of proliferation-associated nucleolar protein p120 expression in prostate adenocarcinoma: a comparison with cyclins A and B1, Ki-67, proliferating cell nuclear antigen, and p34cdc2. *Cancer* **85**: 1569–1576

Pardee AB (1989) G1 events and regulation of cell proliferation. *Science* **246**: 603–608

Pines J (1995) Cyclins, CDKs and cancer. *Semin Cancer Biol* **6**: 63–72

Sherr CJ (1996) Cancer cell cycles. *Science* **274**: 1672–1677

Wang J, Chenivesse X, Henglein B and Brechot C (1990) Hepatitis B virus integration in a cyclin A gene in a hepatocellular carcinoma. *Nature* **343**: 555–557

Wilson GD (1998) Tpot and head and neck cancer: where are we now? *Anticancer Res* **18**: 4801–4805

*© 2000 Cancer Research Campaign*
